# Five‐point liquid rhinoplasty: Results from a retrospective analysis of a novel standardized technique and considerations on safety

**DOI:** 10.1111/jocd.15326

**Published:** 2022-09-20

**Authors:** Leonard Nenad Josipovic, Sonja Sattler, Thilo L. Schenck, Gerhard Sattler

**Affiliations:** ^1^ Rosenpark Klinik Darmstadt Germany; ^2^ Division of Hand, Plastic and Aesthetic Surgery University Hospital, Ludwig Maximilian Universität München Munich Germany

**Keywords:** filler, hyaluronic acid, liquid rhinoplasty, nonsurgical rhinoplasty, Vycross

## Abstract

**Background:**

Nonsurgical aesthetic improvement of the nose with hyaluronic acid (HA) fillers is becoming increasingly popular but comes with important safety considerations.

**Aims:**

To assess the safety and effectiveness of the standardized “five‐point liquid rhinoplasty” approach.

**Methods:**

This was a retrospective, single‐center analysis of consecutive adult patients undergoing nonsurgical aesthetic treatment of the nose. All had one of the three main indications (insufficient nasal projection, nasal hump, or deep glabella) and were injected using HA fillers with high elastic modulus. Treatments were given using some or all of the injection points of the “five‐point liquid rhinoplasty” technique: P1 (nasal tip, 0.2–0.3 ml supracartilaginous); P2 (nasal root, 0.1–0.2 ml supraperiostal); P3 (cartilaginous dorsum, 0.1–0.2 ml supracartilaginous/supraperiostal); P4 (subnasal, 0.1–0.2 ml supraperiostal); and P5 (nasal alar, 0.1–0.2 ml per side supraperiostal). The risk of complications was minimized by injecting deep and staying in the midline as far as possible.

**Results:**

Twenty patients were enrolled (*n* = 15 female [75%]; mean age: 37.8 ± 11.7 years). The mean volume of filler used was 0.66 ± 0.35 ml per patient. Nineteen subjects (95%) said they were “very satisfied” with results, and the physician was also “very satisfied” with outcomes in 19 patients (95%). Two individuals experienced hematoma, which was managed conservatively; there were no other significant complications.

**Conclusions:**

The “five‐point liquid rhinoplasty” is a simple and effective method designed to maximize safety. It offers a good alternative to surgery in selected patients.

## 1 INTRODUCTION

The use of minimally invasive aesthetic procedures continues to increase across the world—including in Germany.^1^, ^2^ This trend is driven primarily by the growing popularity of injectable hyaluronic acid (HA) fillers and botulinum neurotoxin type A formulations. However, the nose remains an area of the face that is often considered to be more amenable to surgery rather than nonsurgical approaches. Indeed, an estimate from the International Society of Aesthetic Plastic Surgery suggested that the number of surgical rhinoplasties performed across the world increased by 13% in 2019 relative to 2018.^1^

The advent of novel HA fillers with greater cohesivity and increased elastic modulus (G′) may shift the balance toward nonsurgical approaches. These enhanced rheological properties are crucial for reducing product spread following injection into the nose—where high skin tension over prominent bone and cartilage can lead to significant compressive forces (and thus loss of projection when using less cohesive products).^3^ With these novel fillers, effective nonsurgical rhinoplasty could be more feasible for more patients.

Effective, minimally invasive treatment of the nose with injectables may offer a number of important advantages over surgical approaches, including reduced patient downtime, removal of the need for anesthesia, lower complication rates, high levels of reversibility (using hyaluronidase), and decreased upfront costs for the patient.^4^, ^5^, ^6^

Nonetheless, not all defects of the nose can be addressed using HA fillers. Appropriate patient selection remains crucial. In addition, there are important safety concerns when injecting this area, including rare but potentially life‐changing complications, such as tissue necrosis or blindness caused by accidental intra‐arterial injection of filler.^7^, ^8^ Greater systematization of the approach to treatment may be important not only for minimizing the risk of such complications, but also for improving the reproducibility of results—thereby helping to optimize patient satisfaction.

We have developed a standardized “five‐point liquid rhinoplasty” aimed at safe treatment of the most relevant aesthetic areas using HA fillers that have high G′ (and ideally also high cohesivity). Most importantly, this method focuses injections along the midline and into deep anatomical layers where the risk of vascular compromise may be lowest.^9^, ^10^, ^11^, ^12^ The objective of the present work was to assess the safety and effectiveness of this technique in a cohort of patients treated in routine clinical practice.

## 2 MATERIALS AND METHODS

### 2.1 Study design and subjects

This was a retrospective, single‐center analysis of consecutive eligible patients undergoing nonsurgical aesthetic treatment of the nose using HA fillers between April 2018 and October 2020. All were injected based on the standardized “five‐point liquid rhinoplasty” technique. The study was conducted in accordance with the Declaration of Helsinki and subjects were required to provide written informed consent ahead of treatment.

Included patients were healthy females and males aged ≥18 years. All were assessed as having one of the following three main indications for HA filler injection in the nose: insufficient projection (i.e., a need to raise the nasal tip); a nasal hump; or a deep glabella. Patients were excluded if they had undergone previous aesthetic surgery to the nose or if their nasal defect was too extreme to be treated with a filler (e.g., a particularly large nasal hump). Individuals with a known allergy to any of the study products, or who were pregnant or lactating, were also excluded.

### 2.2 Procedures

All patients were treated in the nose using Juvéderm® Voluma (Allergan), Juvéderm® Volux (Allergan), or Belotero® Volume (Merz). Treatments were given using some or all of the five injection points of the standardized “five‐point liquid rhinoplasty” technique (Figure 1). Specifically, these were P1 (nasal tip; 0.2–0.3 ml supracartilaginous), P2 (nasal root; 0.1–0.2 ml supraperiostal), P3 (cartilaginous dorsum; 0.1–0.2 ml supracartilaginous/supraperiostal), P4 (subnasal; 0.1–0.2 ml supraperiostal), and P5 (nasal alar; 0.1–0.2 ml per side supraperiostal). Typical recommended filler quantities are provided in parentheses above, although there was some allowance for using slightly more when required based on clinical judgment. All injections were made slowly with low pressure, using a 27G needle and a bolus technique, and injection sites were disinfected ahead of the procedure. Aspiration (with a 3‐second pause) was considered mandatory.

**FIGURE 1:**
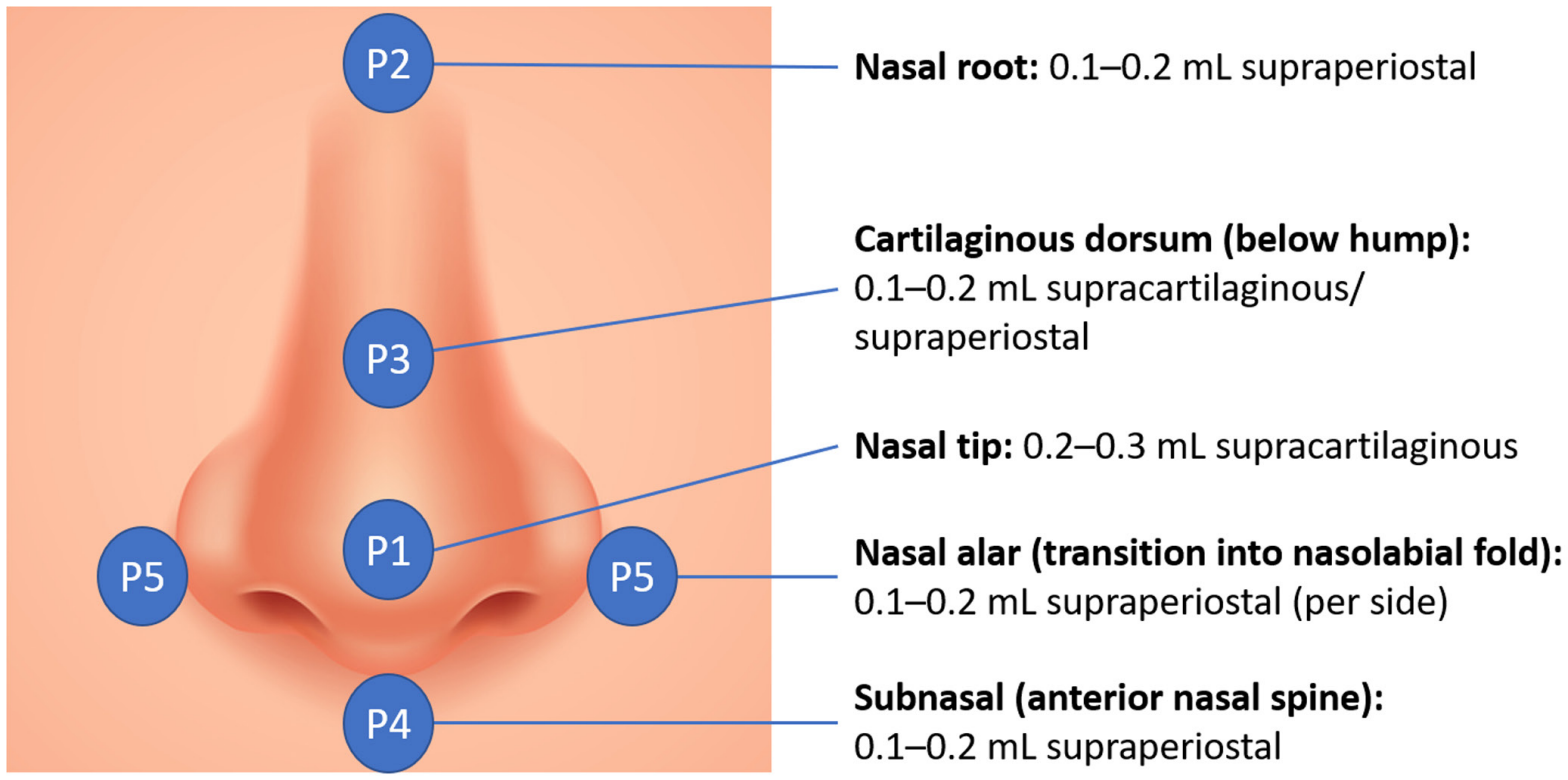
Five‐point liquid rhinoplasty: Injection scheme

### 2.3 Assessments

Age, sex, ethnicity, and the main indication for treatment of the nose were recorded at baseline for all included subjects. The product used and the injection volume at each of the points P1–P5 were also documented. Standardized patient photographs were captured prior to treatment and immediately afterwards. Patients were followed up for a minimum of 6 months.

Patient and physician satisfaction with treatment were assessed immediately after injection on a four‐point scale: very satisfied; satisfied; neither satisfied nor dissatisfied; or dissatisfied.

Complications were evaluated during treatment and follow up.

### 2.4 Statistical analyses

Descriptive statistics are provided throughout, including mean, standard deviation and range for continuous variables, and frequency and percentage for categorical variables.

## 3 RESULTS

### 3.1 Baseline characteristics and treatments

Twenty patients were included in the present analysis, of whom 15 (75%) were female and 5 (25%) were male (Table 1). The mean age was 37.8 ± 11.7 years (range: 23–62 years). All were Caucasian.

**TABLE 1:** Treatment volumes

Main indication	Patients	Female	Male	Total
All	Patients, *n* (%)	15 (75)	5 (25)	20 (100)
	Treatment volume, ml, mean ± SD (range)	0.63 ± 0.35 (0.3–1.6)	0.74 ± 0.33 (0.2–1.0)	0.66 ± 0.35 (0.2–1.6)
Insufficient nasal projection	Patients, *n* (%)	9 (45)	2 (10)	11 (55)
	Treatment volume, ml, mean ± SD (range)	0.58 ± 0.38 (0.3–1.6)	0.60 ± 0.40 (0.2–1.0)	0.58 ± 0.39 (0.2–1.6)
Nasal hump	Patients, *n* (%)	6 (30)	2 (10)	8 (40)
	Treatment volume, ml, mean ± SD (range)	0.72 ± 0.27 (0.3–1.0)	0.75 ± 0.25 (0.5–1.0)	0.73 ± 0.27 (0.3–1.0)
Deep glabella	Patients, *n* (%)	0 (0)	1 (5)	1 (5)
	Treatment volume, ml, mean ± SD (range)	0 (0)	1.00 ± 0.00 (1.0–1.0)	1.00 ± 0.00 (1.0–1.0)

Abbreviation: SD, standard deviation.

The main indications for treatment of the nose were: insufficient nasal projection, *n* = 11 (55%); nasal hump, *n* = 8 (40%); or deep glabella, *n* = 1 (5%). Most patients were treated using Juvéderm Voluma (*n* = 13; 65%) or Juvéderm Volux (*n* = 6; 30%), although 1 individual (5%) received Belotero Volume.

The mean volume of filler used was 0.66 ± 0.35 ml per patient (range: 0.2–1.6 ml). This was broadly similar for both females and males (Table 1). However, the mean treatment volume was slightly lower for the treatment of insufficient nasal projection (0.58 ± 0.39 ml) compared with nasal hump (0.73 ± 0.27 ml).

With regard to injection points, all 20 patients were treated at P1 (Figure 2A), using mean quantities of ≥0.2 ml of HA filler, irrespective of indication (Figure 2B). Other injection points were utilized as needed. Most patients with insufficient nasal projection were treated at P2 (*n* = 8/11; 73%; mean quantity: 0.15 ml) and P3 (*n* = 6/11; 55%; mean quantity: 0.15 ml). Among individuals with a nasal hump, the majority were treated at P2 (*n* = 8/8; 100%; mean quantity: 0.18 ml), P3 (*n* = 6/8; 75%; mean quantity: 0.12 ml), and P4 (*n* = 5/8; 63%; mean quantity: 0.16 ml). The single patient with a deep glabella was treated at all 5 injection points.

**FIGURE 2:**
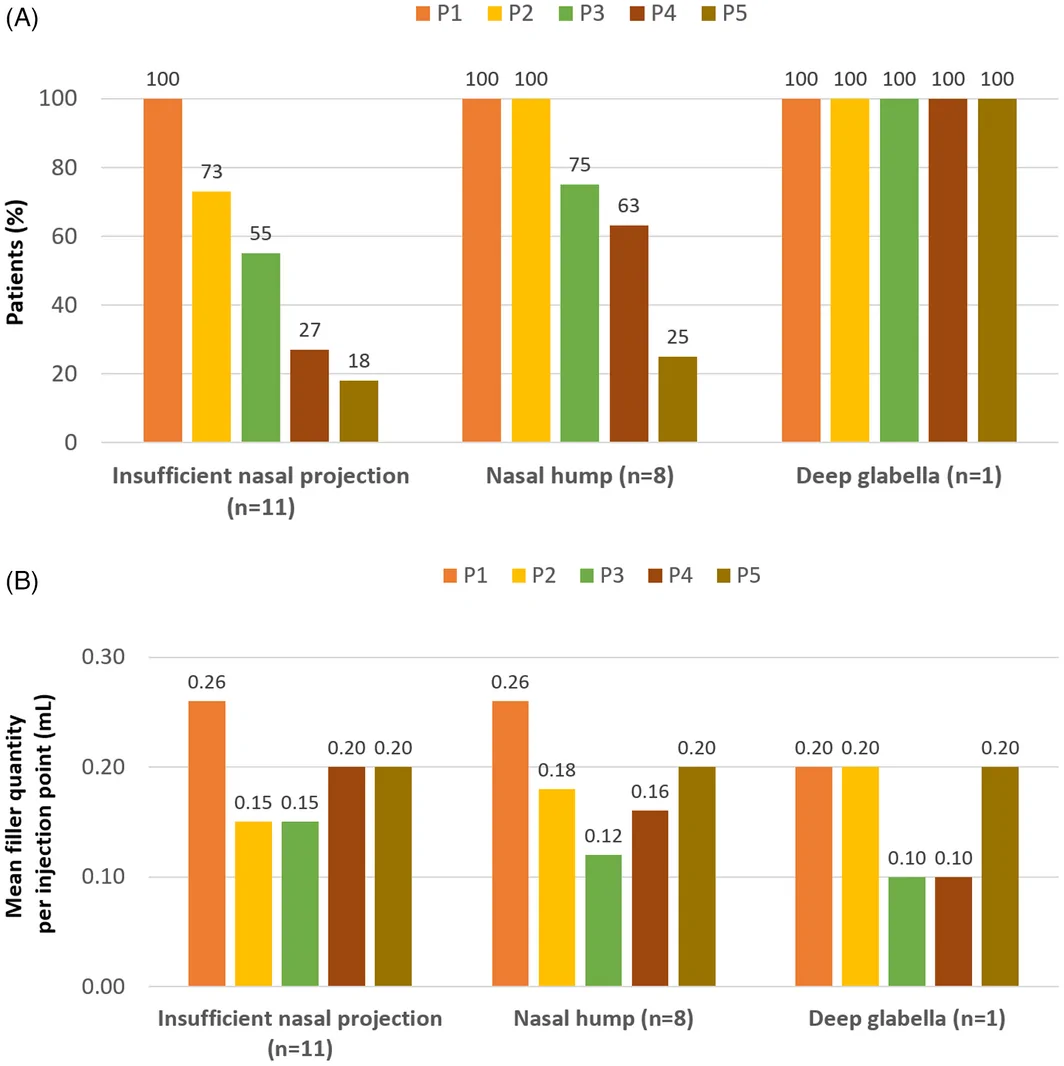
Injection points. Frequency of use of each injection point (A) and the mean quantity of filler used (B) for each of the main indications for treatment. Filler quantity at P5 is “per side”

### 3.2 Satisfaction with treatment

All patients and the injecting physician were asked to assess their satisfaction with the outcome directly after treatment using a four‐point scale (Figure 3). Nineteen patients (95%) said that they were “very satisfied”; the remaining individual (5%) was “neither satisfied nor dissatisfied”. The physician was “very satisfied” with the outcome achieved with 19 patients (95%) and “satisfied” in the other case (5%). Example photographs taken before and after treatment are provided in Figures 4, 5, 6.

**FIGURE 3:**
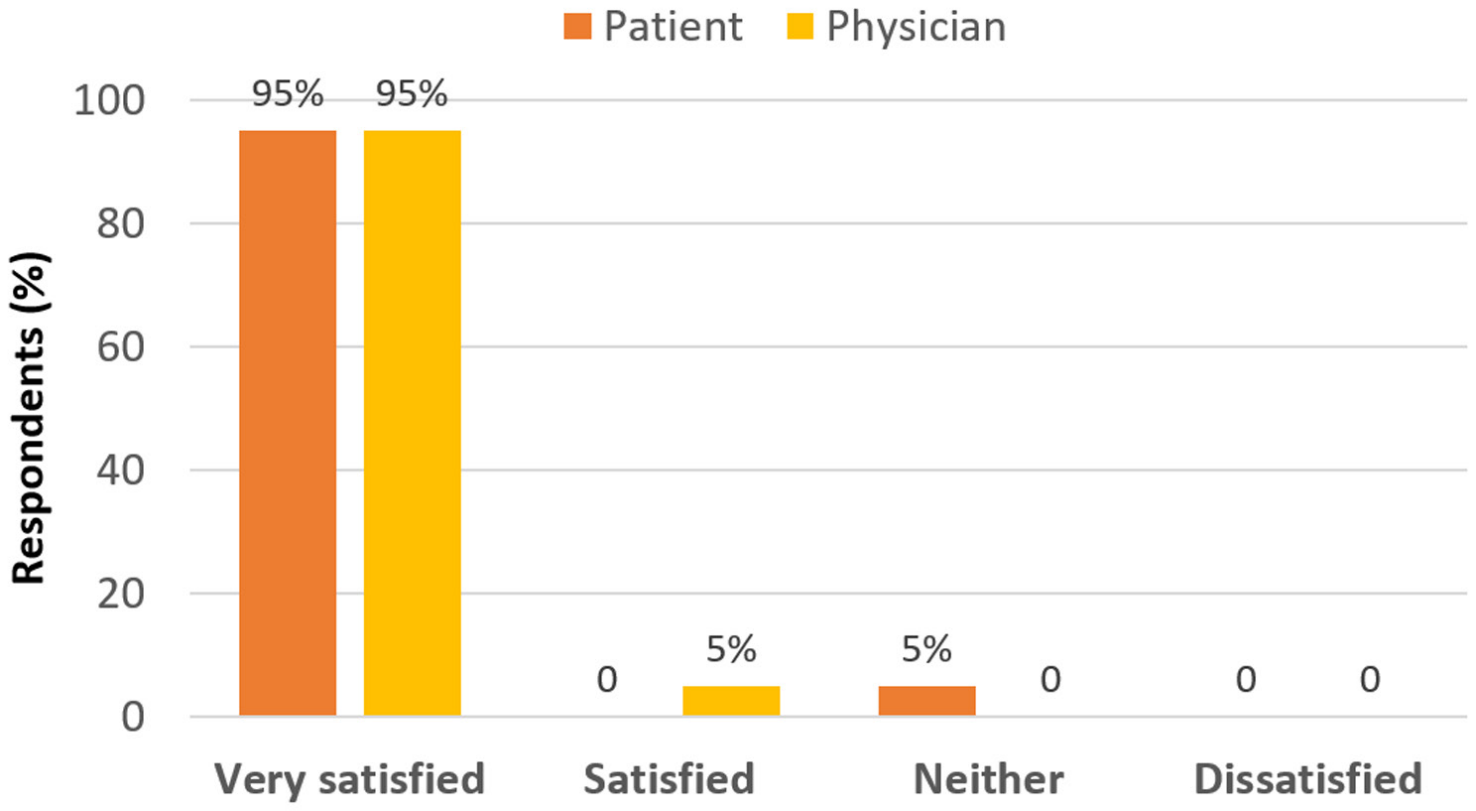
Patient and physician satisfaction with treatment outcome. Satisfaction was assessed on a four‐point scale: very satisfied; satisfied; neither satisfied nor dissatisfied; or dissatisfied. *N* = 20

**FIGURE 4:**
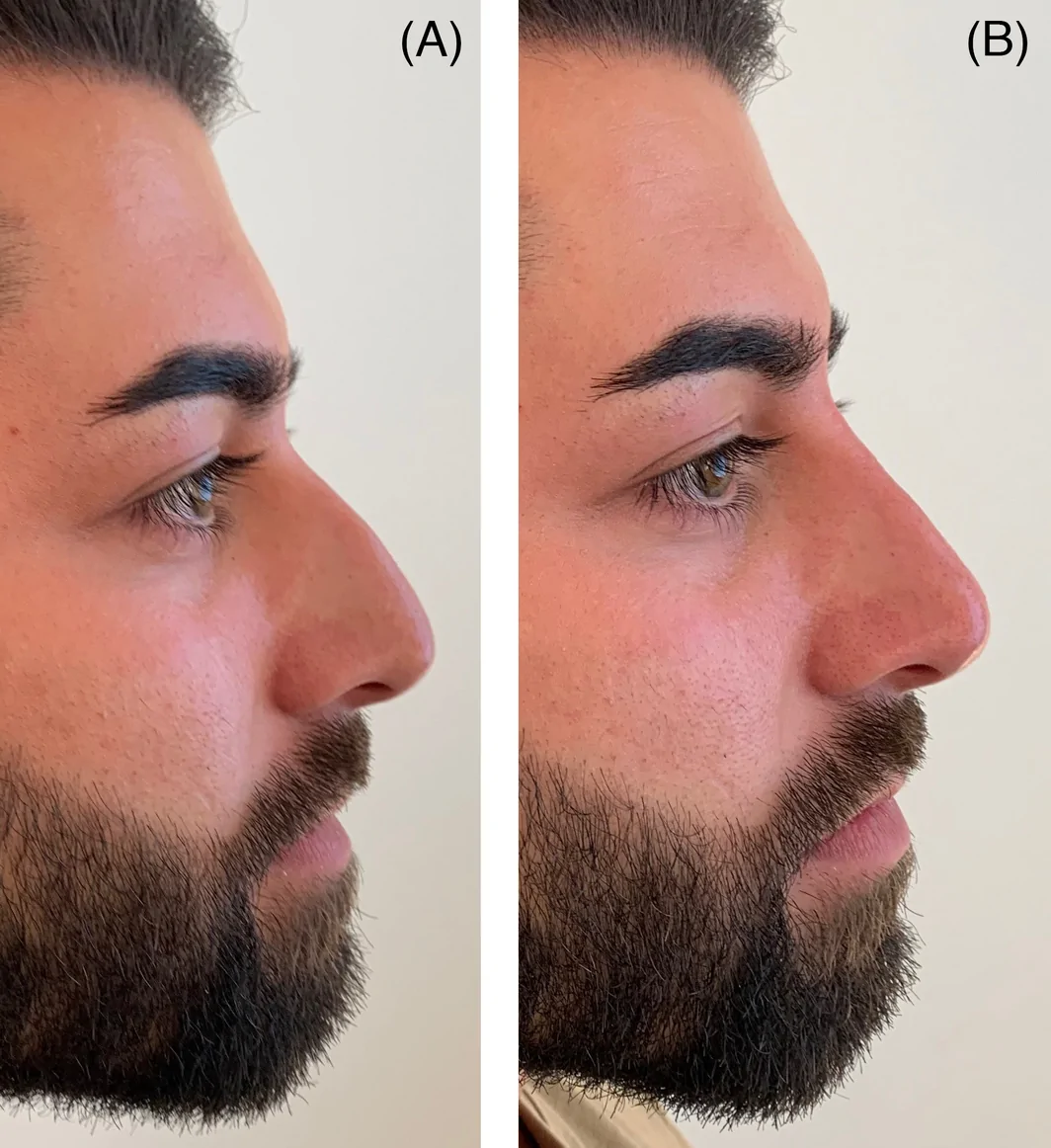
Aesthetic treatment of the nose using the five‐point liquid rhinoplasty. A 26‐year‐old male before and immediately after treatment of the nose using 1.0 ml of Juvéderm Voluma. The main indication for treatment was a nasal hump. He received 0.2 ml of filler at point P1, 0.2 ml at P2, 0.1 ml at P3, 0.1 ml at P4, and 0.2 ml per side at point P5

**FIGURE 5:**
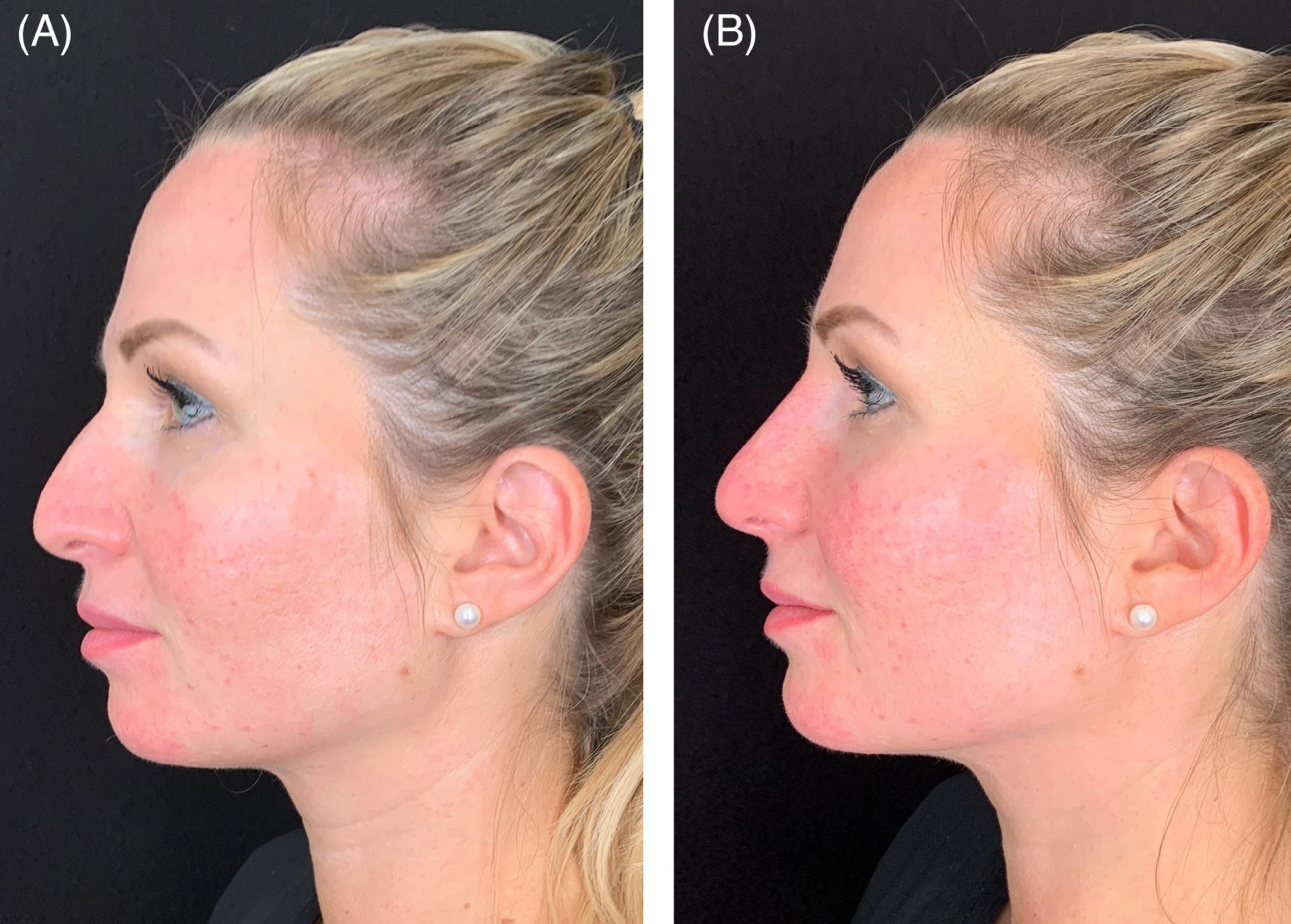
Aesthetic treatment of the nose using the five‐point liquid rhinoplasty. A 35‐year‐old female before and immediately after treatment of the nose using 1.0 ml of Volux. The main indication for treatment was insufficient projection (although she also had a nasal hump and a deep glabella). She received 0.2 ml of filler at point P1, 0.2 ml at P2, 0.1 ml at P3, 0.1 ml at P4, and 0.2 ml per side at point P5

**FIGURE 6:**
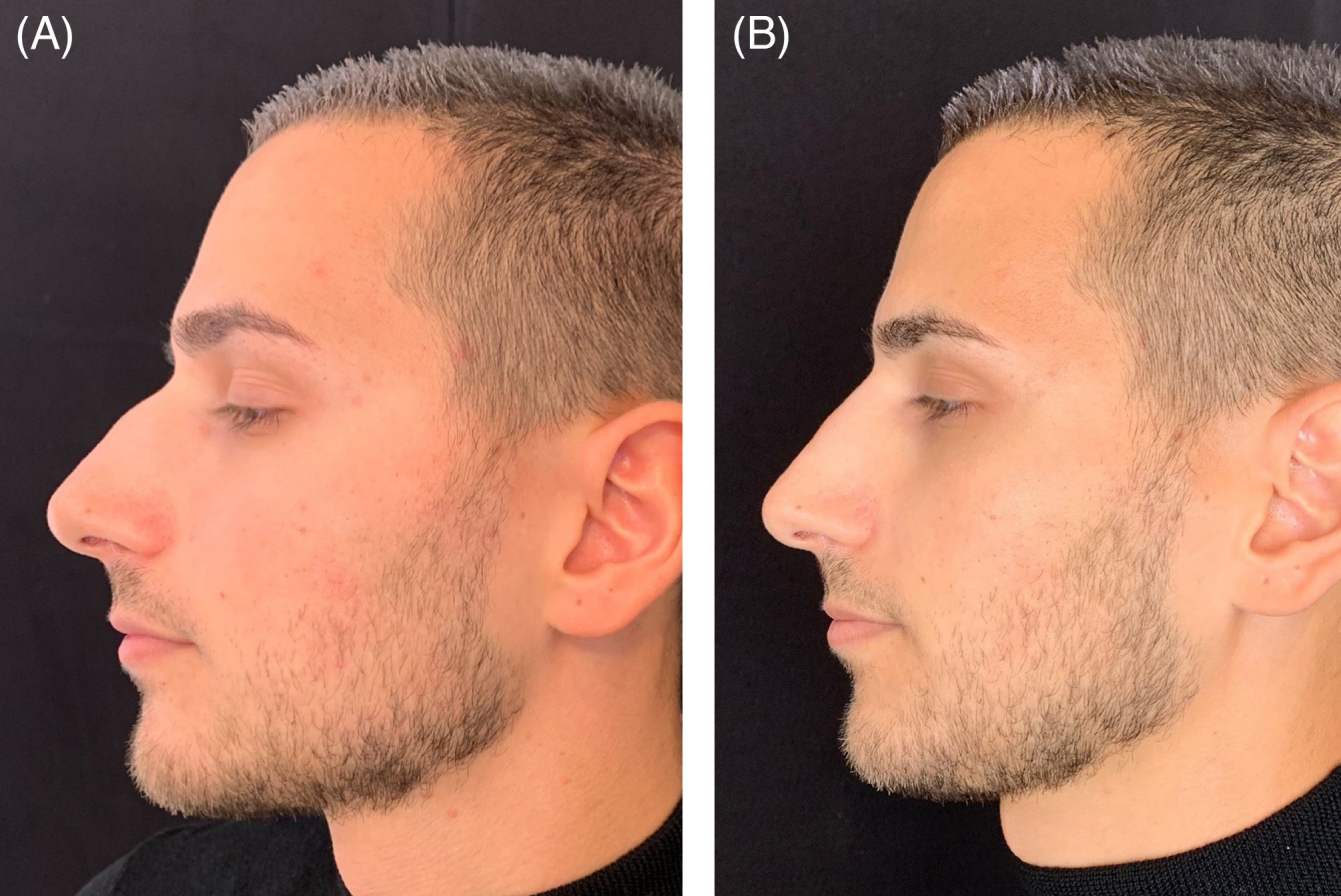
Aesthetic treatment of the nose using the five‐point liquid rhinoplasty. A 23‐year‐old male patient before and immediately after treatment of the nose using 1.0 ml of Volux. The main indication for treatment was a nasal hump. He received 0.2 ml of filler at point P1, 0.2 ml at P2, 0.1 ml at P3, 0.1 ml at P4, and 0.2 ml per side at point P5

### 3.3 Complications

All patients had some swelling after treatment, but this was considered to be an expected sequela of nasal injection rather than a complication. Two patients (10%) experienced hematoma requiring no active treatment, although both were observed to ensure that the filler had not been injected into a vessel. One individual (5%) experienced some overfilling, which was easily managed by extirpation of the excess volume with a needle. There were no instances of bruising, bumps, infection, or skin necrosis.

## 4 DISCUSSION

This study provides preliminary evidence of the safety and efficacy of nonsurgical rhinoplasty using the “five‐point liquid rhinoplasty” technique. The method was developed primarily to minimize the risk of significant complications, and no such events were observed in the present patient group. Two key elements underlying the safety of the technique are: (i) the injection depth (to the bone/cartilage) and (ii) the position of four of the five injection points within the midline (P5 being the only exception).

With regard to injection depth, selection of the optimal plane may be crucial in reducing the risk of vascular compromise. Anatomical analyses have found that the location of facial arteries within a given plane can often vary substantially between individuals but that there are fewer variations in their depth—suggesting that these vessels “respect” their anatomical plane more than their position within that plane.^9^, ^10^ Injection in the supraperiosteal plane or above the cartilage may minimize the probability of damaging a vascular structure and therefore reduce the risk of serious complications. Others have also recommended that the nose should be injected at this level.^13^, ^14^, ^15^, ^16^

Injecting in the midline could also help to minimize the risk of vascular damage. For example, in the glabellar region, a recent ultrasound analysis showed that the supratrochlear and supraorbital arteries were never located below the midline, although their positions moved on frowning, demonstrating that this is an area of highly mobile soft tissue.^12^ Similarly, ultrasound assessment of individuals undergoing filler‐based augmentation of the nasal dorsum found that the course of the dorsal nasal artery was lateral in 80% of patients,^11^ suggesting that medial injection may be safer. Nonetheless, that still left a significant minority (20%) whose dorsal nasal artery was found at the midline of the nose. Caution is therefore warranted, and practitioners must take other precautions when injecting this area. We favor the use of aspiration as an essential safety checkpoint; although this practice remains controversial in some quarters,^17^ recent expert consensus papers have noted that injectors should at least consider it.^5^, ^18^ In practice, we pause for around 3 seconds before continuing with the procedure. A recent systematic review demonstrated an association between aspiration time and the G′ and cohesivity of the filler, as well as the cross‐sectional area of the needle lumen.^19^ Hence, this process should be tailored to the specific procedure. In addition, all procedures in the current analysis were performed using a needle, which may increase the precision of filler placement relative to a cannula.^20^ More generally, practitioners should always remain vigilant for signs and symptoms of possible intravascular occlusion, such as pain and blanching of the skin.^5^, ^18^

Importantly, the “five‐point liquid rhinoplasty” was developed not only with the aim of improving safety but also for providing effective cosmetic improvement, and this has been demonstrated in our results. Levels of patient and physician satisfaction were high, with both parties being “very satisfied” with 19 of the 20 procedures (95%). The high satisfaction levels we observed align with those achieved in previous studies. Indeed, a recent systematic review of eight trials of nonsurgical rhinoplasty with HA fillers demonstrated patient satisfaction levels of 98.2% (*n* = 628/639 patients) in the weeks following injection.^21^

In the single case in which there was not universal satisfaction with the outcome, the patient experienced some overfilling. This was easily managed by extirpation of the excess volume with a needle. The patient had received quantities of filler greater than our standard volumes (Figure 1), and we no longer recommend this practice.

Our technique may also help to reduce the risk of the nose appearing excessively large after filler treatment, which could be an issue in some cases, particularly if P1 were treated in isolation. The use of points P4 and P5 in selected patients can modify the nose ventrally, and hence mitigate this risk.

To reduce product spreading, all procedures were performed using HA fillers with high G′ (and ideally also with high cohesivity).^22^, ^23^ In the nose, these specific rheological properties are more important than the particular product range used, and the “five‐point liquid rhinoplasty” should be feasible using any appropriate HA filler with proven safety and efficacy. Nonetheless, 19 of the 20 patients in the present analysis received fillers from the Vycross range (Voluma or Volux). In our practice, Volux is now becoming the product of choice for injecting the nose. It has the highest HA concentration within the Vycross range (25 mg/ml), and comparative preclinical analyses suggest that it also has the highest cohesivity and G′.^3^, ^22^, ^24^ In a recent Italian study, Volux was found to be safe and effective for treatment of the nose,^15^ and ultrasound analysis has shown that it integrates completely into the tissue within 30 days of implantation.^25^ Although products with high cohesivity and high G′ have potential downsides when used in the nose—such as a possible increased risk of elevated pressure within the surrounding vessels that could reduce skin perfusion—we believe that these are outweighed by the upsides already described.

It must be stressed that filler‐based approaches to aesthetic improvement of the nose should only be attempted by experienced injectors with relevant training, and that they are not appropriate for all patients. Hence, surgical rhinoplasty still has an important place in the aesthetic medicine armamentarium. In the present analysis, the use of HA fillers was reserved for specific indications: insufficient nasal projection; nasal hump; and deep glabella.

This study has some important limitations. In particular, it was a retrospective, single‐center analysis based on a modest number of patients. A prospective, randomized trial would of course be valuable. Furthermore, all of the enrolled subjects were Caucasian. Given the substantial differences in nasal anatomy that are often observed in other racial groups—such as black and Asian populations^26^, ^27^—we must be cautious in extrapolating our data to these individuals.

## 5 CONCLUSIONS

Increasing numbers of patients are presenting in clinic requesting nonsurgical aesthetic improvement of their nose. The advent of novel HA fillers with optimized rheological properties has made these procedures more feasible for more patients than ever before. Our data suggest that the “five‐point liquid rhinoplasty” technique offers an effective and simple‐to‐use method designed to maximize safety, with high levels of resulting patient satisfaction. It therefore provides a good alternative to surgical rhinoplasty in carefully selected individuals.

## AUTHORS' CONTRIBUTIONS

L.N.J. designed the study, performed the research, and analyzed the data. All authors were involved in data interpretation. All authors contributed to writing the paper and approved the final content.

## CONFLICT OF INTEREST

The authors report no conflicts of interest to declare.

## Data Availability

The data that support the findings of this study are available from the corresponding author upon reasonable request.
